# Mouthwash Based on Ozonated Olive Oil in Caries Prevention: A Preliminary In-Vitro Study

**DOI:** 10.3390/ijerph17239106

**Published:** 2020-12-06

**Authors:** Gianna Maria Nardi, Sara Fais, Cinzia Casu, Marta Mazur, Roberto Di Giorgio, Roberta Grassi, Felice Roberto Grassi, Germano Orrù

**Affiliations:** 1 Department of Dental and Maxillofacial Sciences, Sapienza University of Rome, 00161 Rome, Italy; giannamaria.nardi@uniroma1.it (G.M.N.); roberto.digiorgio@uniroma1.it (R.D.G.); 2 Department of Surgical Sciences, Oral Biotechnology Laboratory, University of Cagliari, 09124 Cagliari, Italy; sarafais79@gmail.com (S.F.); ginzia.85@hotmail.it (C.C.); orru@unica.it (G.O.); 3 Department of Biomedical Sciences, University of Sassari, 07100 Sassari, Italy; grassi.roberta93@gmail.com; 4 Department of Basic Medical Sciences, Neurosciences and Sense Organs, University of Bari Aldo Moro, 70122 Bari, Italy; feliceroberto.grassi@uniba.it; 5 National Research Council of Italy, ISPA-CNR, 07100 Sassari, Italy

**Keywords:** ozonated olive oil, *S. mutans*, mouthwash, caries

## Abstract

(1) Background: Ozone (O3) proved to oxidize organic and inorganic compounds, and its efficacy against bacteria, viruses and fungi plasma membranes was of interest. Ozone vehicle can be a gaseous form, ozonated water or ozonized oil. The aim of this in-vitro study was to evaluate the efficacy of ozonated olive oil against *Streptococcus mutans*. (2) Methods: Two different commercial mouthwashes were tested: Ialozon Blu (IB) (Gemavip, Cagliari, Italy), with ozonated olive oil, and Ialozon Rose (IR) (Gemavip, Cagliari, Italy), with ozonated olive oil, hyaluronic acid and vitamin E. All formulates were analyzed in a dilution range from 2- to 256-folds in saline solution, as to reproduce the salivary dilution. *Streptococcus mutans* CIP103220 strain was used for the antimicrobial susceptibility test, and the Kirby–Bauer inhibition method was performed to evaluate the Minimum Inhibitory (MIC), Minimum Bactericidal (MBC), and Minimum Biofilm Inhibitory Concentration (MBIC). (3) Results: Both formulates showed the same antimicrobial activity. MIC, MBC, and MBIC were observed for dilution factors of 1/32, 1/8 and 1/8, respectively. The mean value of inhibition zone diameter was 16.5 mm for IB, and 18 mm for IR. (4) Conclusions: The results suggested that ozonized olive oil formulates were able to inactivate *Streptococcus mutans* avoiding the salivary dilution effect in the oral cavity.

## 1. Introduction

Ozone (O_3_) is a gas composed of three oxygen atoms and it oxidizes organic and inorganic compounds with an immediate effect, impacting, too, the bacteria, viruses and fungi plasma membranes, causing their elimination [1]. Ozone is applied to tissues in different forms: gaseous mixture of oxygen and ozone, ozonated water, or conveyed in the form of oils [2].

Many studies have focused on antimicrobial activity of ozonated products, with evidence of efficacy in gaseous form, against Gram-positive and Gram-negative bacteria, such as *Porphyromonas endodontalis* and *Porphyromonas gingivalis* [2].

Ozonated water showed excellent results against Candida Albicans, reducing Colony Forming Unit (CFU) more effectively than a topical antifungal [3]. The ozonated oil has been tested in the treatment of different pathologies such as joint and skin diseases and it has been proposed in different fields in dentistry: bone regeneration, remineralization of white spots, root canal retreatments due to persistent pathogens, disinfection of periodontal pockets, whitening, management and resolution of hypersensitivity and temporomandibular joint pain [4,5].

Several clinical and in-vitro studies documented the effectiveness of ozone therapy in the management and prevention of caries. Anti-caries activity of ozone was showed in a study by Holmes aiming to arrest and remineralize root caries in a randomized 18-month clinical trial with daily topical use [6]. Baysan and Lynch evaluated the effect of ozone on oral microbiome and primary root caries, with an evident reduction in the microorganisms present in root caries at 5.5 months of follow-up [7]. Gaseous form of ozone was used in a clinical trial to investigate its effect on non-cavitated initial occlusal fissure caries compared with untreated contra-lateral control lesions in a high-risk caries population. At 3 months of follow-up, results revealed that the ozone-treated lesions showed significantly more caries reversal, or reduced caries progression, than the untreated control lesions. The authors concluded that gaseous ozone improved non-cavitated initial fissure caries in patients at high caries risk over a 3-month period [8].

Both gaseous and aqueous ozone were evaluated in in-vitro studies to assess their efficacy in caries management. Ozonated water in a study by Nagayoshi et al. was shown to strongly inhibit the accumulation of in-vitro dental plaque, with great efficacy against Gram-positive and negative oral microorganisms. In fact, *Streptococcus mutans* remarkably decreased in the experimental dental plaque after 10 s of exposure to 4 mg/L ozonated water [9]. Moreover, Polydorou et al. evaluated in an in-vitro study the antibacterial effect of gas ozone, with an evident antibacterial effect on *S. mutans* at 4 and 8 weeks of follow-up [10].

Mouthwashes based on ozonated olive oil were tested in several recent studies. A very new randomized double-blind and controlled clinical study compared the therapeutic action of ozonated olive oil with other therapies in periodontitis patients. The analyzed treatments were; causal therapy with root planning; root planning and ozonated oil; ozonated oil alone; chlorhexidine—the golden standard in periodontal therapy. The results showed that ozonated olive oil, together with planning or alone, was effective in reducing periodontal indices [11].

A very recent systematic review summarized the current state of evidence on the mouthwashes with anti-biofilm and cariostatic properties. The results showed that the most commonly studied active agents were chlorhexidine gluconate, essential oil, cetylpyridinium chloride and fluoride (amine fluoride, stannous fluoride, sodium fluoride, sodium monofluorophosphate) and that those containing fluoride were recommended, based on evidence, as part of a caries-preventive strategy for individuals at high risk of caries [12].

Studies on mucogingival surgery showed that ozonated olive oil promoted the healing of grafts, and when compared with a control group treated with placebo oil, demonstrated a significantly more favorable prognosis of the graft [13,14].

Up to now, no study evaluated the effect of a mouthwash based on ozonated olive oil on caries, neither in in-vivo nor in in-vitro study.

The aim of this in-vitro study was to evaluate the efficacy of ozonated olive oil against the main causative agent of caries, *S. mutans*.

## 2. Materials and Methods

### 2.1. Study Design

**Figure 1:**
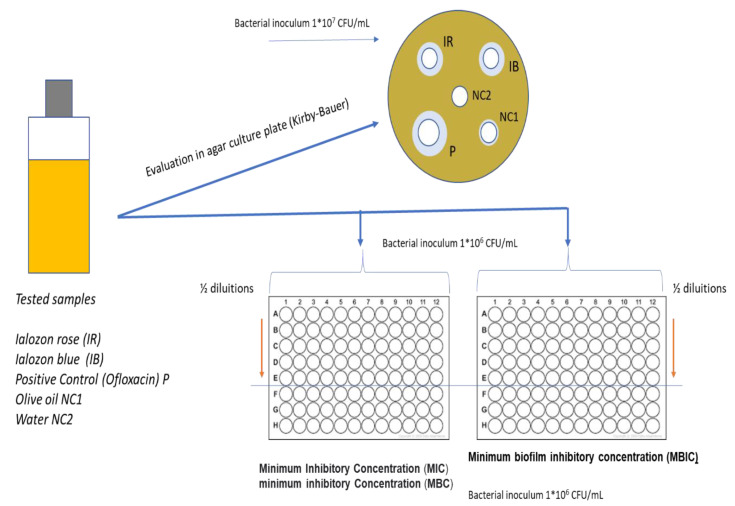
Schematic representation of the study design.

The analysis was based on an in-vitro evaluation by using a study design previously described in the literature and reported in the CLSI (Clinical Laboratory Standards Institute) and in the EUCAST (European Committee for Antimicrobial Susceptibility Testing) [15,16,17] (Figure 1).

The microbiological analyses were performed through three different operative steps: (i) initial antibacterial evaluation in the agar plate; (ii) measurement of the minimal inhibition concentration and minimal bactericidal concentration; (iii) evaluation of the formulate activity against bacterial biofilm formation. Ofloxacin disc of 5 μg (Oxoid) and a well of 50 µL of water were used as positive and negative controls, respectively (Figure 1).

### 2.2. Olive Oil Ozonated Mouthwashes and Negative Control

Two different commercial mouthwashes were tested: (i) Ialozon Blu (Gemavip, Cagliari, Italy), IB, with ozonated olive oil and (ii) Ialozon Rose (Gemavip, Cagliari, Italy), IR, with ozonated olive oil, hyaluronic acid and vitamin E. As a negative control, extra virgin olive oil (S. Giuliano, Alghero, Italy) was used. All formulates were analyzed in a dilution range from 50 to 3.1% in saline solution, with the aim to reproduce the salivary dilution in the mouth during an oral rinse. 

### 2.3. Bacterial Strain and Microbiological Analysis

A strain of *S. mutans*, CIP103220 (Collection de l’Institut Pasteur) was used for the antimicrobial susceptibility test.

For this in-vitro experiment, the Kirby–Bauer inhibition method was used to evaluate the antibacterial activity of the ozonated olive-oil-based mouthwashes. The test involved 5 phases:

1. Preparation of culture medium with 15 mL of Scheadler-agar medium (Microbiol, Uta, Cagliari) at 55 °C, in a 90 mm diameter Petri dish. The plate was characterized by 1 sterile iron rivet with a diameter of Ø 10 mm and a thickness of 2 mm which was removed after the agar solidification;
2. Standardized bacterial inoculum (5 × 10^7^ CFU/mL) was seeded on the surface of the plate using a sterile swab;
3. Insertion of the material to be tested: 50 µL of Ialozon mouthwash with different dilution for each dish (ozonated olive oil, Gemavip, Cagliari, Italy) was dispensed through a micropipette;
4. Incubation of the plate with *S. mutans* at 37 °C with 5% CO_2_ for 24 h;
5. After incubation, the diameter of the possible zone of inhibition was measured with a caliper for each dish, and dilutions of the tested substances, which determined an inhibition zone greater than 10 mm in diameter, were recorded.

The Minimum Inhibitory (MIC) and Minimum Bactericidal Concentration (MBC) were assessed by the micro-dilution method while the Minimum Biofilm Inhibitory Concentration (MBIC) was evaluated following the modified protocol described by Montana University Canter for Biofilm Engineering, as well as following previous published protocols [18].

Briefly, each formulation serially diluted (1:2) in Schaedler broth (Microbiol Uta, Cagliari, Italy), ranging from 50% to 0.02%, was tested in sterile NuncTM MicrowellTM 96-well microplates (Thermo Fisher Scientific, Rodano (MI), Italy). Then, a Suspension of *S. mutans* in Schaedler Broth, 1 × 10^8^ CFU/mL, corresponding to 0.5 McFarland index) was inoculated into each well to obtain a final inoculum concentration of 10^6^ CFU/mL. 

After 48 h at 37 °C in 5% CO_2_ atmosphere, the plates were read with a microplate reader at 620 nm (Multiskan FC, Thermofisher, MA, USA). The MIC was the lowest concentration of an antimicrobial that inhibited the visible growth (absence of turbidity).

To determine the MBC, 100 µL of the dilution representing the MIC and at least two of the more concentrated formulations were plated in Schaedler agar at 37 °C, and 5% CO_2_; after 24 h, the colony-forming units (CFUs) were enumerated. The MBC was the lowest concentration able to effectively reduce the yeast growth (99.5%). The ability of these formulations, to inhibit the biofilm formation was evaluated following the crystal-violet staining protocol, as previously reported [18]. Inoculum and the formulation dilution procedures were the same as those already described for the MIC and MBC experiments. In this context, the minimum biofilm inhibitory concentration (MBIC) represented the lowest concentration able to interfere with biofilm formation. In other words, after the *S. mutans* biofilm staining using crystal violet, the MBIC represented the concentration of drug able to show an absorbance comparable to the negative control—the sample without bacteria; this represented the blank for the spectrophotometric analysis. This is used to eliminate the signal due to the medium during the absorbance reading.

### 2.4. Statistical Analysis

Each experiment was repeated three times, with a sample size of three for each dilution. For the same concentration, all values that showed a standard deviation (SD) within ±10% of the mean value were considered significant. Social Science Statistics web calculator (test calculator for 2 independent means) with p value assessed at *p* < 0.05, was used to compare the differences between the tested formulations.

## 3. Results

**Figure 2:**
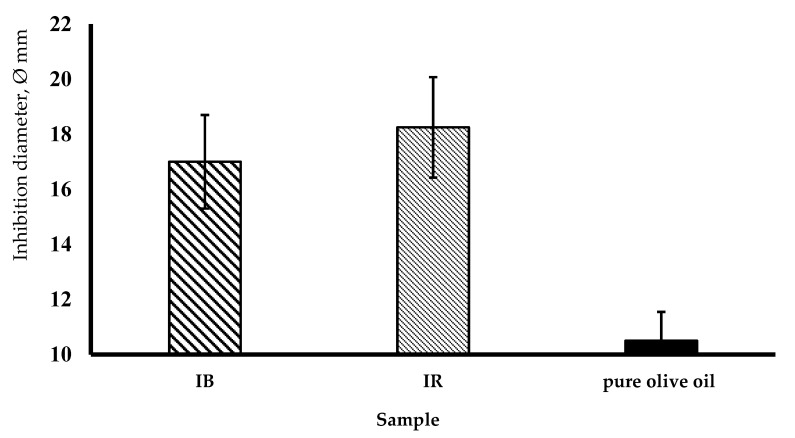
The mean value of inhibition diameters observed for Ialozon Blu (IB) and for Ialozon Rose (IR) and negative control (extra virgin olive oil) (*p* < 0.05 between ozone olive oil formulates and not ozonated oil).

In the first instance, following the Kirby–Bauer diffusion method, both commercial formulates showed a comparable antimicrobial activity. The mean value of inhibition diameter was 16.5 mm for Ialozon Blu, and 18 mm for Ialozon Rose. The difference between the two commercial mouthwashes, in the inhibition diameter, was not significant (*p* > 0.05). While, the negative control (pure olive oil) showed minimal antimicrobial activity against *S. mutans*, (Ø = 11 mm) (Figure 2).

**Figure 3:**
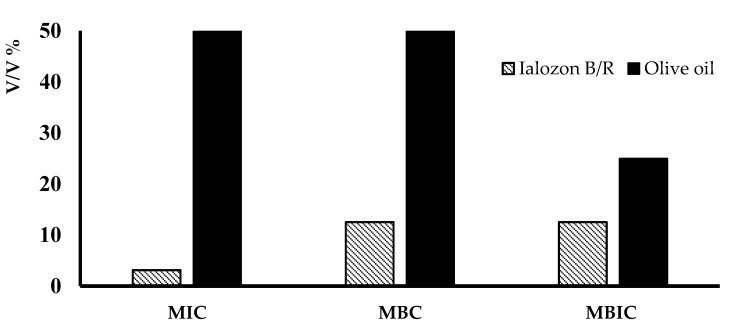
Minimum Inhibitory Concentration (MIC), Minimum Bactericidal Concentration (MBC), and Minimum Biofilm Inhibitory Concentration (MBIC), evaluated for ozonized olive-oil-based products, Ialozon Blu or Rose (B/R) and pure olive oil (control), *p* < 0.05.

MIC, MBC, and MBIC were observed for dilutions factors of 1/32, 1/8 and 1/8, corresponding to 3.1%, 12.5%, 12.5% (volume percent), respectively, for both formulates, while pure olive oil (control) showed MIC, MBC and MBIC of 50%, >50%, 25%, respectively (Figure 3). The results showed that these formulates were able to inactivate the sessile and planktonic form of *S. mutans* avoiding the dilution effect. This indicated a good inhibition of ozonated oil in comparison to control; in fact, by using a concentration of 12.5% of oil-ozone formulates, a 99% inhibition of growth (MBC) was observed, while with commercial olive oil, the inhibition of growth was 2% in the mean, *p* < 0.05.

**Figure 4:**
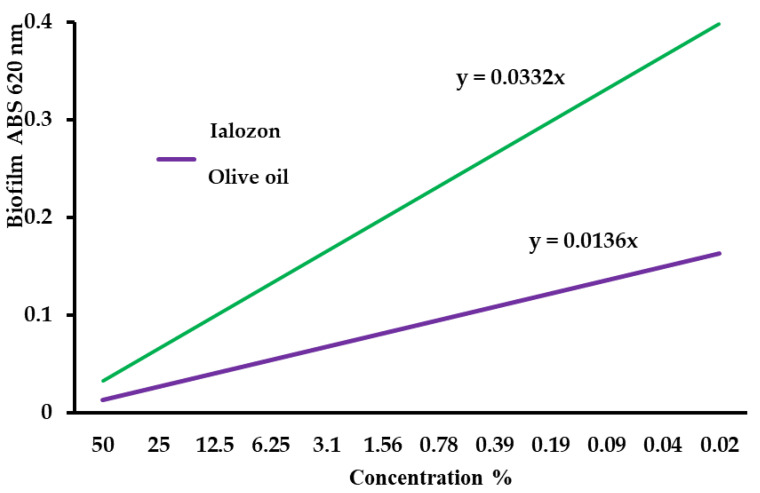
Linear trend curves, indicating the biofilm increase rate in comparison to a decrease in the formulates concentration, observed for ozonized olive oil products (Ialozon), and pure olive oil. The difference observed between curve slope values in the two formulates indicates the major activity in biofilm inhibition due to ozone addition to olive oil. The figure was obtained from the mean values of the biofilm for each formulate.

Figure 4 represents the curves of residual biofilm evaluated with different % concentration of ozonized olive oil products (Ialozon) and pure olive oil (control). The graph suggests that the effect of ozone addition to the olive oil matrix could determine an effective *S. mutans* biofilm reduction as already demonstrated with the previous experiments (Figure 1 and Figure 2).

## 4. Discussion

This in-vitro study assessed for the first time the antibacterial effect on *S. mutans* of ozonated olive oil mouthwashes. The results showed that both tested formulates were able to inactivate *S. mutans*, in comparison with a control, represented by extra virgin olive oil.

*S. mutans* is an acidogenic bacteria associated with the onset of caries in primary and secondary dentition. Dental caries result from a shift in the biofilm community. Acidogenic bacteria colonize the tooth surface and cause enamel demineralization in the presence of fermentable carbohydrates. Dental caries is in fact defined as a disease with multifactorial etiology.

The *S. mutans* cariogenicity is characterized by: (i) adherence to enamel surface; (ii) production of acidic metabolites; (iii) the capacity to build up glycogen reserves; (iv) the capacity to synthesize extracellular polysaccharides (EPSs) [19]. In patients with high caries risk, the mechanical removal of biofilm by at-home oral hygiene procedures may often be insufficient. Adjunctive use of antimicrobial agents helps to control plaque formation and composition by: (i) reducing the overall rate of plaque accumulation; (ii) reducing partially or totally the existing plaque; (iii) inhibiting the growth of those species mostly linked to the disease; (iv) inhibiting EPS production. Triclosan, xylitol and chlorhexidine have been used for the last 40 years as antibacterial topical agents. Triclosan showed an unfavorable biocompatibility and was banned in 2016 by the USA Food and Drug Administration from soap formulations [20]. Chlorhexidine is nowadays the gold standard for topical oral antimicrobial agents; however, its use for a prolonged period of time is not recommended due to side effects such as tooth discoloration, taste alterations and oral desquamation [5].

The biocompatibility of ozone was evaluated by in-vivo and in-vitro studies. No cytotoxic signs were observed, and aqueous ozone showed the highest biocompatibility among the tested antiseptics. Moreover, a very recent clinical study on a mouthwash based on ozonated olive oil used for a period of 3 months showed no side effects correlated with its daily use [5].

The current study provides new data on the efficacy of ozonated-based oral formulations against the principal pathogen of dental caries. A previous review article on ozone therapy aimed to assess its effectiveness in the management and prevention of caries; the results showed that the analyzed clinical in-vivo studies provided higher evidence of ozone efficacy in treating and preventing dental caries when compared to the outcomes of the in-vitro analyzed studies. The review conclusions claimed for more evidence from in-vitro studies for management and prevention of caries [21].

This current in-vitro study assessed for the very first time the antimicrobial effect of two mouthwashes based on ozonated olive oil. Both formulates showed a comparable antimicrobial activity. MIC, MBC, and MBIC were observed for an in-volume concentration of 3.1%, 12.5%, 12.5%, respectively. The biofilm reduction speed, as showed in Figure 4, was more effective by using the ozonized olive oil in comparison with the control. Moreover, the results showed that by increasing the ozonized product % concentration, the efficacy on biofilm reduction was three times higher compared to the control. The present results showed that these formulates were able to inactivate the sessile and planktonic form of *S. mutans*, avoiding the dilution effect. *S. mutans* is often associated with dental plaque, forming highly organized microbial communities. The ability to form biofilm has been considered as one of the virulence factors for Mutans Streptococci, including *S. mutans*.

Moreover, the comparison of the two ozonized commercial oil formulations did not show significant differences; in fact, the presence of hyaluronic acid and vitamin E in the Ialozon Rose product was unable to create an additive antibacterial activity (*p* < 0.05). This behavior could be explained due to an inadequate concentration of these agents or to the unexpected oxidative reaction of these compounds with ozone. Further experiments are necessary to investigate this result.

The result of the current study is in accordance with previous publications regarding the ozone activity against Gram-positive bacteria. Wang et al. assessed that the inhibition effect of *S. mutans* depended on the ozone concentration and the treatment time. For example, 2.73 mg/L ozone in water for 45 min was able to kill this pathogen [22].

Ialozon Blu is a mouthwash with ozonated olive oil, while Ialozon Rose is within ozonated olive oil and, in addition, hyaluronic acid and vitamin E. Olive oil is a central ingredient of the Mediterranean diet and it is rich in oleic acid and phenolic compounds [23]. Olive oil showed to be useful for enhancing fluoride inhibition of extracellular polysaccharide (EPS) formation by *S. mutans* and preventing biofilm formation [24]. Therefore, olive oil, as a single ingredient, may be useful in promoting oral health both in the prophylaxis of dental caries and periodontitis [25]. In the present study, the Ialozon Blu and Ialozon Rose formulations’ olive oil is ozonized and the effect against *S. mutans* is evident.

Moreover, previous study highlighted topical ozone as an effective enamel remineralizing agent [26]. A recent study compared the antibacterial activity against *S. mutans* of ozonated water with 0.20% chlorhexidine [27]. The study included 46 pediatric patients divided into two groups, all with intraoral concentrations of *S. mutans* greater than 10^5^ CFU, evaluated with salivary samples at baseline. After 14 days with daily chlorhexidine and ozonated water mouthwashes, the levels of CFU were reduced in both groups, but more in the group with ozonated water [27]. However, until today, no study has tested ozonated olive oil against *S. mutans*. The current in-vitro study provides new evidence on the efficacy of ozonated olive oil mouthwashes against the principal pathogen of dental caries. Dental caries is a multifactorial process, and implementing prevention is very important. In both the pediatric and adult population, compliance to tooth brushing and at-home oral hygiene procedures might be difficult to achieve on a daily basis. The use of new ozonated olive oil mouthwashes could be an additional preventive tool for the at-home oral hygiene procedure. It shows great efficacy on *S. mutans* within a safety profile with its use over time.

Further research is needed to assess the efficacy of mouthwashes based on ozonated olive oil against *S. mutans* by in-vitro and in-vivo clinical studies in high-caries-risk children and adult populations and in subjects with oral breathing that need orthodontic therapy [28,29,30].

## 5. Conclusions

In conclusion, this study showed the antibacterial and antibiofilm activity against *S. Mutans* of two formulates with ozonated olive oil. The study highlighted the safety of these mouthwashes for daily use, although additional data on their efficacy are needed.

## Links/Footnotes

CLSI’s Subcommittee on Antimicrobial Susceptibility Testing (AST)	https://clsi.org/meetings/ast/
The European Committee on Antimicrobial Susceptibility Testing—EUCAST	https://eucast.org/
Social Science Statistics web calculator	https://www.socscistatistics.com/tests/studentttest/default2.aspx
